# How do we increase uptake of tamoxifen and other anti-estrogens for breast cancer prevention?

**DOI:** 10.1038/s41523-017-0021-y

**Published:** 2017-05-19

**Authors:** Katherine D. Crew, Kathy S. Albain, Dawn L. Hershman, Joseph M. Unger, Shelly S. Lo

**Affiliations:** 1 Columbia University Medical Center, Herbert Irving Comprehensive Cancer Center, New York, NY USA; 2 Loyola University Chicago Stritch School of Medicine, Cardinal Bernardin Cancer Center, Maywood, IL USA; 3 SWOG Statistical Center, Fred Hutchinson Cancer Research Center, Seattle, WA USA

## Abstract

Several randomized controlled trials of anti-estrogens, such as tamoxifen and aromatase inhibitors, have demonstrated up to a 50–65% decrease in breast cancerincidence among high-risk women. Approximately 15% of women, age 35–79 years, in the U.S. meet criteria for breast cancer preventive therapies, but uptake of these medications remain low. Explanations for this low uptake includelack of awareness of breast cancer risk status, insufficient knowledge about breast cancer preventive therapies among patients and physicians, and toxicity concerns. Increasing acceptance of pharmacologic breast cancer prevention will require effective communication of breast cancer risk, accurate representation about the potential benefits and side effects of anti-estrogens, targeting-specific high-risk populations most likely to benefit from preventive therapy, and minimizing the side effects of current anti-estrogens with novel administration and dosing options. One strategy to improve the uptake of chemoprevention strategies is to consider lessons learned from the use of drugs to prevent other chronic conditions, such as cardiovascular disease. Enhancing uptake and adherence to anti-estrogens for primary prevention holds promise for significantly reducing breast cancer incidence, however, this will require a significant change in our current clinical practice and stronger advocacy and awareness at the national level.

## Introduction

Breast cancer is the most common malignancy among women in the U.S. and worldwide.^1^ Although breast cancer mortality has decreased over the past few decades, incidence continues to rise particularly in developing countries.^2, 3^ Unlike cardiovascular disease prevention, there are a limited number of oral medications available for cancer prevention. Based upon results from several randomized controlled trials (Table 1),^4^ anti-estrogens, such as selective estrogen receptor modulators (SERMs) and aromatase inhibitors (AIs), have been associated with a 50–65% relative risk reduction in invasive breast cancer among high-risk women.^5–9^ Due to the strength of this evidence, the U.S. Preventive Services Task Force (USPSTF), American Society for Clinical Oncology (ASCO), National Comprehensive Cancer Network (NCCN), and the National Institute for Health and Care Excellence (NICE) recommend that clinicians discuss preventive therapy with high-risk women.^10–13^ About 10 million women in the U.S. are eligible for breast cancer preventive therapy,^14^ but fewer than 10% of high-risk women offered an anti-estrogen for primary prevention agree to take it.^15, 16^ Because breast cancer risk assessment is not routinely conducted in the primary care setting, many women and their physicians may be unaware of their risk of developing breast cancer and that preventive options are available. These options include lifestyle modifications, such as reduction of alcohol consumption, increasing exercise, and maintaining a healthy body weight, and pharmacologic options. Many primary care providers (PCPs) may feel uncomfortable prescribing a medication that is commonly prescribed by cancer specialists. The perception is that since these medications are used to treat cancer, they may have significant side effects.^15, 17^ The commonly used phrase of “chemoprevention” for the use of anti-estrogens for primary prevention often has negative connotations, however, “preventive therapy” may be more acceptable.^18^ Further research is needed to determine how to better educate and communicate with physicians and women about breast cancer risk, healthy lifestyle, and preventive therapy options available.

**Table 1:** Updated results from major randomized controlled trials of selective estrogen receptor modulators (*SERMs*) and aromatase inhibitors (*AIs*) for breast cancer preventive therapy

Trial	No. of participants	Study population	Agents	Breast cancer risk reduction RR or HR (95% CI)	Toxicities compared to controls (Rate per 1000)
BCPT, 2005 (ref. 5)	13,388	Age ≥ 35 years, 5-yr Gail risk score > 1.66% if age 35-59 years or age ≥ 60 years or LCIS	Tamoxifen 20 mg/day × 5 years vs. placebo	0.57 (0.46–0.70)	Endometrial cancer (2.24 vs. 0.68)
					Stroke (1.75 vs. 1.23)
					Thromboembolism (1.90 vs. 1.16)
IBIS-I, 2014 (ref. 6)	7154	Age 35–70 years, tenfold risk if age 35–39 years or fourfold risk if age 40–44 years or twofold risk if age 45–70 years	Tamoxifen 20 mg/day × 5 years vs. placebo	0.71 (0.60–0.83)	Endometrial cancer (8.10 vs. 5.59)
					Stroke (8.38 vs. 7.83)
					Thromboembolism (13.97 vs. 8.11)
STAR, 2015 (refs 7, 100)	19,490	Age ≥ 35 years, postmenopausal, 5-year Gail risk score > 1.66%	Raloxifene 60 mg/day vs. Tamoxifen 20 mg/day × 5 years	1.19 (1.04–1.37)*	Endometrial cancer (1.23 vs. 2.25)
					Thromboembolism (2.47 vs. 3.30)
					Cataracts (11.69 vs. 14.58)
MAP.3, 2011 (ref. 8)	4560	Age ≥ 35 years, postmenopausal, 5-year Gail risk score > 1.66% if age 35–59 years or age ≥ 60 years or AH, LCIS, DCIS with mastectomy	Exemestane 25 mg/day × 5 years vs. placebo	0.35 (0.18–0.70)	Fractures (66.51 vs. 63.60)
					Cardiovascular events (47.32 vs. 49.37)
IBIS-II, 2014 (ref. 9)	3864	Age 40–70 years, postmenopausal, fourfold risk if age 40–44 years or twofold risk if age 45–59 years or 1.5-fold risk if age 60–70	Anastrozole 1 mg/day × 5 years vs. placebo	0.47 (0.32–0.68)	Fractures (85.42 vs. 76.65)
					Cardiovascular events (66.67 vs. 48.87)

Adapted from Crew ASCO Educational Book, 2015 with permission from the American Society of Clinical Oncology

*AH* atypical hyperplasia, *BCPT* Breast Cancer Prevention Trial, *CI* confidence interval, *DCIS* ductal carcinoma in situ, *HR* hazard ratio, *IBIS* International Breast cancer Intervention Study, *LCIS* lobular carcinoma in situ, *MAP* mammary prevention, *RR* relative risk, *STAR* Study of Tamoxifen and Raloxifene

*Comparing raloxifene to tamoxifen

### Who is eligible for breast cancer preventive therapy?

The Gail breast cancer risk assessment tool (BCRAT),^19^ easily accessible at no cost on-line, is the most commonly used tool to estimate a woman’s risk of developing breast cancer. This tool was used to determine eligibility for breast cancer preventive therapy in the North American prevention trials. It incorporates current age, age at menarche, age at first live birth, race/ethnicity, first-degree family history of breast cancer, and the presence of benign breast disease, including atypical hyperplasia. Eligibility for chemoprevention included women aged ≥ 60 years or those with a 5-year invasive breast cancer risk greater than1.67% or lifetime risk greater than 20%. The European prevention trials used the Tyrer-Cuzick model,^20^ which incorporates genetic and non-genetic breast cancer risk factors including a more extensive family history, to determine eligibility for pharmacologic prevention. For the International Breast cancer Intervention Studies of tamoxifen (IBIS-I) and anastrozole (IBIS-II), women who had a 10-year risk of breast cancer according to the Tyrer-Cuzick model of 5% or greater were eligible to enroll.^6, 9^ Both the Gail and Tyrer-Cuzick models have been cited as inadequate for risk assessment. The Tyrer-Cuzick model overestimates risk of cancer in women with breast atypia.^21^ The Gail model has been well-validated at the population level,^19^ and has been updated for more sensitive risk estimates in African American^22^ and Asian American populations.^23^ However, few studies have used this model in Hispanic populations.^24, 25^ Other breast cancer risk models, such as Tyrer-Cuzick, BRCAPRO, and BOADICEA (Breast and Ovarian Analysis of Disease Incidence and Carrier Estimation Algorithm), may be used to estimate risk in women with a strong family history of breast or ovarian cancer.^26^

Women with high-risk benign breast lesions, such as atypical ductal hyperplasia, atypical lobular hyperplasia, and lobular carcinoma in situ (LCIS), have up to a 4- to 10-fold increased risk of breast cancer compared to women with non-proliferative breast disease.^27^ Long-term studies indicate that women with atypical hyperplasia or LCIS have a greater than 30% lifetime risk of breast cancer.^28, 29^ Due to the high estrogen receptor (ER) expression in benign breast disease,^30^ these women appear to derive a greater benefit from anti-estrogensin terms of breast cancer risk reduction compared to other high-risk populations. The subgroup of women with atypical hyperplasia enrolled in the chemoprevention trials had a 41–79% relative risk reduction in breast cancer incidence.^5, 6, 8, 31, 32^ Since uptake of breast cancer preventive therapy remains low among these women,^33^ this is an important high-risk population to specifically target for use of anti-estrogens for primary prevention.

A new and emerging group of women who are at increased risk of developing breast cancer are those with a gene mutation that is associated with a moderately increased risk of breast cancer. The ability to test for multiple genes (panel testing) has revealed a group of women who may be at moderately elevated risk of developing breast cancer. *CHEK2, ATM, CDH1, NBN, NF1, PALB2*, among others, are now being identified. It is unclear if chemoprevention would be efficacious in this population of women.

### What are the potential benefits of breast cancer preventive therapy?

The Breast Cancer Prevention Trial (BCPT) showed that 5 years of tamoxifen reduced breast cancer incidence in high-risk women by 49% (ref. 34). Other trials of tamoxifen for primary prevention confirmed a 30–40% breast cancer risk reduction compared to placebo.^35–38^ Based upon long-term follow-up in the IBIS-I, the protective effect of tamoxifen for breast cancer risk reduction persisted after discontinuation of therapy.^6^ Another SERM, raloxifene, has been shown to reduce breast cancer incidence and improve bone health among postmenopausal women.^39, 40^ Although initial results from the Study of Tamoxifen and Raloxifene (STAR) trial suggested that raloxifene may be inferior to tamoxifen in lowering noninvasive breast cancer risk among high-risk postmenopausal women,^41^ updated results demonstrated that raloxifene was 25% less effective than tamoxifen for lowering invasive breast cancer risk, but had a more favorable side effect profile with fewer uterine cancers and thromboembolic events.^7^ The results of these trials led to the approval by the U.S. Food and Drug Administration (FDA) of tamoxifen in 1998 and raloxifene in 2007 for the primary prevention of breast cancer among high-risk women.

MAP.3 (Mammary Prevention Trial-3) confirmed the efficacy of AIs for the primary prevention of breast cancer among high-risk postmenopausal women. Five years of exemestane was associated with a 65% relative risk reduction in invasive breast cancer incidence compared to placebo.^8^ Similar findings were observed in the IBIS-II trial in high-risk postmenopausal women with a lower incidence of invasive and non-invasive breast cancers seen with anastrozole for 5 years vs. placebo (hazard ratio [HR] 0.47; 95% confidence interval [CI] 0.32–0.68; *p* < 0.0001). Extended hormonal therapy in the adjuvant setting with the AI, letrozole, for up to 10 years compared to 5 years for women with early stage invasive breast cancer has been shown to be more effective at preventing contralateral breast cancers.^42^ This study also brings into question about the optimal duration of AI therapy for breast cancer prevention.

### What are the potential risks of breast cancer preventive therapy?

Limitations of both SERMS and AIs as preventive therapy include: lack of efficacy against ER-negative breast cancers, sparse efficacy data among women with hereditary breast cancer syndromes,^43, 44^ unclear duration of chemoprevention for optimal efficacy, and lack of evidence in reducing breast cancer-specific or overall mortality. The reason why none of the prevention studies have shown an improvement in overall survival is because the primary end point of the prevention studies has been breast cancer incidence. Without demonstrating an improvement in overall survival, some physicians may be reluctant to prescribe a long-term medication with potential for toxicity. Additionally, none of the prevention studies have demonstrated an improvement in quality of life (QOL) for women who choose chemoprevention. However, lowering breast cancer incidence may impact the rising costs of diagnosing, treating, and providing care for breast cancer patients, with annual costs for breast cancer care projected to surpass $20 billion by 2020 (ref. 45). Several cost-effectiveness analyses of tamoxifen and raloxifene for preventive therapy have demonstrated increased quality adjusted life years or cost-savings in select populations.^46–51^

Concerns about rare but serious side effects of tamoxifen, such as endometrial cancer and thromboembolism, are the main reasons for reluctance among high-risk women to take this agent and reservations among physicians to prescribe it.^52–58^ The risk-benefit ratio for tamoxifen varies by age, race, and breast cancer risk.^5^ For example, younger women less than 50 years of age, those with a prior hysterectomy, and at higher risk for breast cancer have a more favorable risk-benefit profile with tamoxifen. Among high-risk premenopausal women, tamoxifen is the only FDA-approved preventive therapy for breast cancer;the risk of uterine cancer and thromboembolism in this population is low.^34^ Among postmenopausal women, if documented to have a normal endometrium at baseline, the Southwest Oncology Group S9630 study reported a low incidence (3–6%) of new benign endometrial abnormalities on tamoxifen with up to 5 years of follow-up.^59^ Based on this study, a baseline normal endometrial sonogram could potentially provide some reassurance to women who might otherwise decline tamoxifen therapy due to fear of endometrial cancer. During tamoxifen therapy, women should undergo yearly examinations by a gynecologist with diagnostic work-ups for postmenopausal vaginal bleeding. Routine pelvic ultrasounds or endometrial biopsies during tamoxifen treatment in asymptomatic women is not recommended, because this has not been shown to be effective.^60^

Raloxifene is associated with a lower rate of of thromboembolism, uterine complaints, and cataracts compared to tamoxifen.^41, 61^ Overall QOL was similar in women receiving tamoxifen and raloxifene in the STAR trial.^61^ Compared to placebo, the AIs are associated with in increased rate of vasomotor symptoms (57% vs. 49%), vaginal dryness (19% vs. 16%), arthralgias (51% vs. 46%), and hypertension (5% vs. 3%).^9^ Osteoporosis, decreased bone density and fractures are another concern with AI use; there was no significant difference in new-onset osteoporosis in the MAP-3 study of exemestane vs. placebo.^7^ In addition, there was no difference in cardiovascular events, second malignancies, or QOL in women receiving exemestane vs. placebo.^8^

To provide a more personalized risk-benefit profile of preventive therapy among high-risk postmenopausal women, a model was developed to weigh the potential risks and benefits of tamoxifen and raloxifene based upon age, race/ethnicity, absolute breast cancer risk, and prior hysterectomy.^62^ The first network meta-analysis of the efficacy and acceptability of preventive therapies for breast cancer from randomized controlled trials found that new-generation SERMs, such as raloxifene, had the best risk-benefit ratio, followed by AIs and tamoxifen.^63^ Therefore, high-risk postmenopausal women have more options for preventive therapy with raloxifene and AIs, which have fewer serious side effects compared to tamoxifen.

Of note, the side effects diminish after completing 5 years of anti-estrogens, but the beneficial aspect in preventing breast cancer endures after drug cessation. Unlike other pharmacologic interventions for cardiovascular disease prevention that require chroniclife-long treatment, breast cancer chemoprevention may be limited to 5 years with long-standing benefits with adverse effects present only during active therapy. Table 2 summarizes the patient populations eligible for chemoprevention and the risks and benefits of SERMs and AIs for primary prevention.

**Table 2:** Summary of the high-risk women eligible for chemoprevention and the risks and benefits of selective estrogen receptor modulators (*SERMs*) and aromatase inhibitors (*AIs*) for primary prevention

Patient population for chemoprevention	Benefits of chemoprevention	Risks of chemoprevention
Eligible women:	Selective estrogen receptor modulators:	Selective estrogen receptor modulators:
• Age ≥ 60 years	• 30–50% relative risk reduction in breast cancer incidence	• Vasomotor symptoms, vaginal symptoms, leg cramps
• Five-year risk of invasive breast cancer ≥ 1.67% according to the Gail model	• 33% relative risk reduction in fractures	• Increased risk of cataracts (tamoxifen)
• Ten-year risk of breast cancer ≥ 5% according to the Tyrer-Cuzick model	• Only effective against estrogen receptor-positive breast cancer	• Increased risk of uterine cancer (tamoxifen)
	• Not associated with an overall survival benefit	• Increased risk of thromboembolism
High-risk women with a favorable risk/benefit profile from chemoprevention:	Aromatase inhibitors:	Aromatase inhibitors:
• Age < 50 years	• 50–65% relative risk reduction in breast cancer incidence	• Vasomotor symptoms, vaginal dryness, arthralgias
• Prior hysterectomy	• Only effective against estrogen receptor-positive breast cancer	• Increased risk of osteoporosis
• Atypical hyperplasia or lobular carcinoma in situ	• Not associated with an overall survival benefit	• Increased risk of hyperlipidemia and hypertension
* • BRCA2* mutation carriers		

### Why is there such low uptake of breast cancer preventive therapy?

According to recent systematic reviews and meta-analyses about uptake and adherence to breast cancer preventive therapy, less than 10% of high-risk women offered an anti-estrogen for primary prevention agree to take it.^15, 16^ Based upon data from 26 studies on uptake of preventive therapies, Smith et al.^16^ reported that uptake was 25.2% among women screened for clinical trials, but only 8.7% in the non-trial settings.^16^ After its FDA approval for primary prevention in 1999, tamoxifen use among women unaffected with breast cancer was 0.2% in 2000 and decreased to 0.08% in 2005 (ref. 33). Similary, raloxifene use also decreased after its FDA approval in 2007 for breast cancer prevention.^41^ It is unclear whether AIs will gain greater acceptance compared to SERMs in the primary prevention setting.

The effectiveness of preventive therapy also depends on patient adherence. In the prevention trials, adherence varied from 64 to 85% (refs 5, 8, 37, 61). Adherence to anti-estrogens in the prevention trials is inferior to that in the adjuvant setting.^64^ In a cohort study, almost half the women receiving tamoxifen prevention (46%) discontinued treatment within 4.5 years.^65^ Factors associated with poor drug adherence include lower socioeconomic class and members of ethnic minorities.^66, 67^ Better understanding of the predictors for poor uptake and adherence will inform the development of targeted interventions that may improve utilization of chemoprevention for high-risk populations.

Most patients and providers may believe that the risks of side effects of chemoprevention may outweigh the potential benefits for breast cancer risk reduction, therefore, use of these medications remains low. Concern about side effects has been associated with lower uptake for primary prevention.^52^ Strategies to minimize side effects of anti-estrogens include developing novel endocrine agents, novel administration including intermittent or lower drug dosing, and alternative drug delivery systems, such as topical chemoprevention agents. Novel SERMs used for osteoporosis, such as arzoxifene,^68^ bazedoxifene,^69^ and lasofoxifene,^70^ show evidence of reducing breast cancer risk with a better side effect profile compared to tamoxifen.^63^ Several studies of low-dose tamoxifen (1–10 mg daily) or intermittent dosing of 10–20 mg every other day or weekly have shown similar biologic effects to daily dosing of tamoxifen 20 mg with fewer adverse effects.^71–78^ Transdermal tamoxifen applied to the breasts, is another novel, and perhaps more acceptable prevention modality.^79^ Two presurgical window-of-opportunity trials in women with invasive and non-invasive breast cancer demonstrated that topical 4-hydroxytamoxifen (4-OHT) was comparable to oral tamoxifen for decreasing tumor proliferation with lower systemic drug levels.^80, 81^ The long-term effects of these alternative strategies for breast cancer prevention are actively being investigated.

In addition to concerns about toxicities, another major barrier to uptake of anti-estrogens for preventive therapy is a robust risk assessment model that accurately estimates patient risk. The Gail model is the most widely used risk assessment model, but its accuracy may be limited by patient ethnicity and consideration of first-degree relatives only when evaluating family history of cancer. The Tyrer-Cuzick model, is not one that is familiar to most PCPs. Only 18% of PCPs report use of the on-line Gail model to estimate a patient’s breast cancer risk.^82^ Different medical specialties vary widely in their use of the Gail model (33–37% for internal medicine and family medicine compared to 60% for gynecology) and ever prescribing anti-estrogens (8–9% for internal medicine and family medicine compared to 30% for gynecology).^83^ Additional barriers to routine breast cancer risk assessment include time constraints, lack of familiarity and comfort prescribing preventive therapy.^84^ Physician recommendation and effective communication strongly influence uptake of preventive therapy for breast cancer.^52, 55, 85^ Providers who were less informed about breast cancer risk-reducing options were less than half as likely to prescribe a SERM than those who felt sufficiently trained.^86^

Critics of chemoprevention cite that ER-positive breast cancer is a curable condition; so why should we subject healthy women to a long-term treatment with potential for adverse effects. At 20 years, the number needed to treat to prevent 1 invasive breast cancer is 29; so that 28 women would unnecessarily undergo treatment to prevent 1 curable cancer.^87^ What is needed even more is chemoprevention for the more difficult to cure ER-negative breast cancers. When diagnosed at an early stage, women with ER-positive breast cancer have an excellent prognosis.

Intervention trials of clinical decision support tools designed to increase uptake of breast cancer preventive therapy targeting both patients and providers have met with limited success. A web-based decision aid called *Guide to Decide*, which informed high-risk postmenopausal women about potential benefits and side effects of chemoprevention yielded only 0.5% uptake of raloxifene and no tamoxifen use.^88^ The *Ready, Set, GO GAIL!* project involved systematic screening of over 5700 women by PCPs using the Gail model.^89^ Among 868 (15.2%) women who met high-risk criteria for breast cancer, 14.7% were referred for specialized risk counseling, 6.4% completed the consultation, and only 2% initiated chemoprevention. The *BreastCARE* intervention used a tablet-based patient intake tool in the primary care setting that produced a tailored breast cancer risk report for patients and their physicians.^90^ In a randomized controlled trial of the *BreastCARE* intervention in 1235 women (age 40–74 years), more women in the intervention arm compared to controls were referred for a high-risk consultation (18.8% vs. 4.1%), however, there was limited discussions about chemoprevention documented in the medical record (1% vs. 0%). Orlando et al.^91^ reported on an implementation trial of the *MyTree* family history screener, which generated tailored risk reports for patients and providers. Of 26 women found to be eligible for preventive therapy, none of them initiated an anti-estrogen for breast cancer prevention.

Studies from specialized risk assessment and counseling clinics reported uptake of preventive therapy ranging from 11 to 58% (refs 52–54, 85, 92–94). Because some community practices may not have access to specialized risk counseling, PCPs may need sufficient knowledge about breast cancer risk and chemoprevention to prescribe antiestrogens to high-risk women. More efficient tools are needed to identify chemoprevention eligible women in a busy clinic, and inform both patients and providers about the potential benefits and side effects of pharmacologic therapy. Table 3 summarizes barriers to chemoprevention and potential strategies to increase uptake among high-risk women.

**Table 3:** Summary of barriers to chemoprevention and strategies to increase chemoprevention uptake among high-risk women

Barriers to chemoprevention uptake	Strategies to increase chemoprevention uptake
• Lack of routine breast cancer risk assessment in the primary care setting	• Integration of breast cancer risk assessment into clinic workflow with built-in risk calculators in the electronic health record
• Lack of knowledge about breast cancer chemoprevention among high-risk women and primary care providers	• Development of clinical decision support tools for breast cancer chemoprevention for patients and primary care providers
• Time constraints during the clinical encounter limiting discussions about chemoprevention	• Activating patients and providers with the use of decision aids outside of the clinical encounter to facilitate shared decision-making
• Competing comorbidities among high-risk women	• Targeting younger, healthier women and high-risk women who are likely to benefit from antiestrogens (e.g., atypical hyperplasia, lobular carcinoma in situ)
• Concern for side effects of antiestrogens	• Minimizing side effects with lower doses, different dosing schedules, and different drug preparations (e.g., topical forms)
• Lack of intermediate biomarkers to predict response to chemoprevention	• Investigation of automated measures of mammographic density, as a predictive biomarker of response to antiestrogens

## Conclusions

Breast cancer preventive therapy with anti-estrogens is efficacious among high-risk women; however, acceptance remains low. Potential strategies to improve uptake of preventive therapy include: (1) Targeting-specific high-risk populations, such as younger women who are at lower risk of serious side effects and women with high-risk benign breast lesions who derive greater benefit from anti-estrogens; (2) Minimizing toxicities with alternative endocrine agents and novel drug administration schedules and drug delivery methods; (3) Enhancing breast cancer risk assessment and informed decision-making about preventive therapy in the primary care setting with decision support tools integrated into clinic workflow.

Cancer prevention is not as well established as cardiovascular disease prevention.^95^ Novel ways of conducting breast cancer risk assessment are needed. Screening mammography visits may represent such a “teachable moment”. As the majority of states in the U.S. now have legislation for mandatory breast density notification, when women receive information about high breast density, which also increases breast cancer risk, this may serve as an opportunity to discuss risk-reducing options with their PCP. Health information technologies such as electronic health records and patient health portals may be a method for collecting data on breast cancer risk and presenting information about chemoprevention, which is integrated into clinic workflow.^4^ Preventive therapy may also be integrated into broader strategies of cancer prevention, such as discussions about lifestyle factors, which influence breast cancer risk, such as obesity, physical activity, and alcohol consumption.

Breast cancer incidence continues to increase and the economic burden of cancer in the U.S. is expected to increase significantly,^96^ due to increasing costs of cancer care.^45, 97–99^ Promoting breast cancer prevention should be a priority that involves novel risk assessment strategies, education of patients, and providers, advocacy, and awareness at the national level.
